# Complementary and Alternative Medicine Use in Musculoskeletal Disorders: Does Medical Skepticism Matter?

**DOI:** 10.2174/1874312900701010005

**Published:** 2007-09-18

**Authors:** Elizabeth K Wiley-Exley, Thelma J Mielenz, Edward C Norton, Leigh F Callahan

**Affiliations:** 1 Department of Health Policy and Administration, University of North Carolina (UNC) School of Public Health, USA; 2 Division of Physical Therapy; Thurston Arthritis Research Center, UNC School of Medicine, USA; 3 Department of Health Policy and Administration, UNC School of Public Health, USA; 4 Thurston Arthritis Research Center, UNC School of Medicine, USA

**Keywords:** Medical skepticism, complementary and alternative medicine, musculoskeletal disorders

## Abstract

*Medical skepticism* is the reservation about the ability of conventional medical care to significantly improve health. Individuals with musculoskeletal disorders seeing specialists usually experience higher levels of disability; therefore it is expected they might be more skeptical of current treatment and thus more likely to try Complementary and Alternative Medicine (CAM). The goal of this study was to define these relationships. These data were drawn from a cross-sectional survey from two cohorts: those seeing specialists (n=1,344) and non-specialists (n=724). Site-level fixed effects logistic regression models were used to test associations between medical skepticism and 10 CAM use categories. Some form of CAM was used by 88% of the sample. Increased skepticism was associated with one CAM category for the non-specialist group and six categories for the specialist group. Increased medical skepticism is associated with CAM use, but medical skepticism is more often associated with CAM use for those seeing specialists.

## INTRODUCTION

Arthritis is the most prevalent chronic condition in the adult population [1, 2] and the most common cause of disability in those 65 and older [3, 4]. Although there are multiple ways to treat arthritis and other musculoskeletal disorders, the efficacy of the treatments vary and leave a significant number of individuals with pain and activity limitations [5–7]. To alleviate discomfort and disability associated with these chronic conditions, approximately 50% of patients with musculoskeletal disorders have reported using complementary and alternative medicines (CAM) [8–10].

Understanding how and why patients choose different health care methods, such as CAM, is important for chronic disease management. Self-management of chronic diseases, such as musculoskeletal disorders, requires years of drug regimens, visits to many doctors, and behavior change. Health care providers and policymakers need to better understand the decision-making processes of patients to facilitate discussions about the disease and treatment choices, but this is often a difficult task. There are a variety of health beliefs, such as medical skepticism, which can influence the interplay between needing and receiving care, but these are often unmeasured or unstudied.

*Medical skepticism* is the reservation or doubt about the ability of conventional medical care to significantly improve health status [11]. Former studies looked at the influence of this variable on the use of conventional medical care [11, 12]. Individuals who are skeptical of health care often forgo health care and insurance coverage and engage in less healthy behaviors [11], which could be detrimental to long-term management of musculoskeletal disorders.

**Fig. (1):**
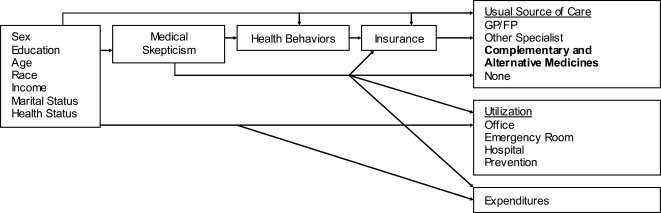
Medical Skepticism variant of the Andersen-Newman behavioral health model modified to account for CAM [11,13,14].

Previous work [11] concerning medical skepticism has been based on a variant of the Andersen-Newman behavioral health model (Fig. **1**) [13, 14]. Andersen’s model helps explain why patients and families use health services and measures equitable access to health care. In the medical skepticism variation of the model, medical skepticism represents a common pathway for the variety of forces that motivate health beliefs and behaviors [11]. In our study, we hope to gain a better understanding of how this health belief would affect the use of CAM. Previous research led us to believe that skepticism reduces the use of conventional care;[11, 12] thus we might expect medical skepticism to increase the use of CAM. There are multiple aspects of this argument, however. It could be that skeptical individuals are more likely to be skeptical of all treatments, thus they might be less likely to use all forms of care. Similarly, individuals who are not skeptical of conventional care may be skeptical of CAM. The variety of relationships which could exist, points to the need for more information about this health belief.

Studies have also shown that different types of practitioners, such as specialists and general internists, serve different types of patients and recommend different forms of care [15, 16]. Individuals with musculoskeletal disorders seeing specialists are usually experiencing higher levels of disability [16], lower levels of functioning and have more severe mus-culoskeletal disorders [15] than individuals seeing non-specialists; therefore it is expected that they might be more skeptical of their current treatment regimens and thus be more interested in trying CAM therapies. The goal of this study is to better define these relationships by looking at the effects of medical skepticism on CAM use in two groups of individuals with musculoskeletal disorders; those being served by family practitioners and those served by specialists. A secondary goal is to report other predictors of CAM use.

## METHODOLOGY

**Population**. A sample of patients with self-report muscu-loskeletal disorders was taken from two cohorts: the North Carolina Family Medicine Research Network (NC-FM-RN) and the musculoskeletal database (MSK). Sloane, Callahan, Kahwati and Mitchel [17] provided more details about the data collection process and the study design for the NC-FM-RN. During 2001, consecutive adult patients at 17 family practice sites in rural and urban areas across North Carolina were asked to complete a self-reported questionnaire to assess health status and socio-demographics. Since 1995, similar health status and demographic questionnaires have been collected for the MSK from individuals attending the University of North Carolina at Chapel Hill rheumatology, orthopaedics, spine center, and sports medicine clinics and private rheumatology offices in Durham, Pinehurst, Sanford, Clyde, Lenoir, and Fayetteville, North Carolina.

Of the eligible participants from the two databases who provided consent, 4,101 reported musculoskeletal disorders. Musculoskeletal disorders were defined as self-report of osteoarthritis (OA), rheumatoid arthritis (RA), fibromyalgia (FM), or chronic joint symptoms (CJS) in the NC-FM-RN. In the MSK, musculoskeletal disorders were self-report physician diagnosis of OA, RA, or FM.

The individuals agreeing to further contact were mailed two survey booklets. The first booklet asked about the patient’s health, health beliefs (including medical skepticism) and use of health care; the second asked patients about use of CAM. Non-respondents were contacted again with a second survey after three weeks and then again by phone if they did not respond to either mail survey. A total of 2,140 patients responded; 72 individuals did not complete the medical skepticism variable and were therefore excluded from the analyses.

**Sample**. Individuals were separated into two groups for analysis: those seeing specialists or those seeing family practitioners (non-specialist) for their musculoskeletal disorders. Individuals were included in our analysis of specialists if they were patients in the rheumatology, orthopedics, spine center, or sports medicine clinics (n=1,048) or if they were in the family practice network but reported seeing a rheuma-tologist or an orthopedic surgeon for their musculoskeletal disorders (n=296), for a total of 1,344 patients. Individuals were included in the non-specialist sample if they were attending one of the family practice sites and not seeing a specialist for their musculoskeletal disorders (n=724).

**Measures**. The primary outcome was use of CAM for musculoskeletal disorders. Participants were asked about their ever or current use of the following categories of CAM in order to deal with musculoskeletal disorders: (1) alternative healthcare providers or therapists; (2) special diets or food plans; (3) vitamins or minerals (not including multivi-tamins, Calcium, Folic Acid or Vitamin D); (4) herbs, mixtures, or other supplements taken by mouth (e.g., glucosa-mine); (5) rubs, lotions, liniments, creams, or oils; (6) body treatments, such as copper bracelets or magnets worn or used; (7) movement activity (e.g., yoga); (8) spiritual activities (e.g., prayer); and (9) relaxation or mind-body activities, including breathing techniques and visualization. Each category included a number of items, and if an individual reported yes for any item, they were scored positively for the category. Each of the nine categories was included as an outcome of interest, as well as one summary outcome to denote whether an individual ever used any of the categories (ever used CAM) for a total of ten outcomes. It should be noted that when this study was completed, glucosamines were still considered as more of an alternative therapy and were not widely prescribed for OA.

The main independent variable of interest was medical skepticism. It is measured by a four-item scale that has been demonstrated to be valid and reliable in similar situations [11]. The four statements are:

1. I can overcome most illnesses without help from a medically trained professional.
2. Home remedies are often better than drugs prescribed by a doctor.
3. If I get sick, it is my own behavior that determines how soon I get well again.
4. I understand my health better than most doctors do.

Each item had a five-point response scale [1 (disagree strongly) - 5 (agree strongly)] assessing attitudes toward medical care, with higher scores indicating more skepticism [11]. The four responses were averaged, and the unweighted mean was used to create the summary score [11, 12].

The other independent variables included in the model were based on the medical skepticism variant of the Ander-sen-Newman behavioral health model:[11, 13, 14] sex, education, age, race and ethnicity, income, marital status and health status. Education was dichotomized into two categories: (1) individuals with some college education, a college degree or a post-graduate education or (2) individuals with a high school degree or less education. Race and ethnicity were categorized as those self-defining as non-Hispanic Caucasian (NHC) (reference category), non-Hispanic Afri-can American (NHAA), and a third category of other and multiple race and ethnicity. Income was not included in the survey; therefore employment was used as a proxy. Employment was dichotomized; the first category included those who were working for full-time pay, working as homemakers, retired, and students. The reference category included participants who self-reported as unemployed, disabled, or other. Marital status was dichotomized: married (reference category) and unmarried. Health status was defined using two measures. The first was based on a self-report of general health: excellent/very good/good health or fair/poor health (reference category). The second measure of health status was a count of fifteen comorbidities, including RA, OA, and FM.

Between 6 and 28 observations were missing on the male, age, and self-reported health variables. Complete case analysis was used for these variables because the observations were assumed to be missing at random and because the missing observations were far less than 5% of the sample. The dummy variable adjustment method was used for missing observations for the college education, race and ethnicity, working, and married variables. This commonly used method allows for retention of the missing observations in the analysis [18]. The missing observations were coded as zero and then a separate dummy variable was created, which represented the missing observations; this variable was then included in the final analyses.

**Statistical Analysis**. A site-level Chamberlain fixed effects multivariate logistic regression analysis was employed to look at how medical skepticism was related to the different CAM therapies, controlling for the variables defined by the Andersen-Newman behavioral health model (Fig. **1**). The analyses were completed using STATA version 8 [19]. The fixed effects represented 13 different sites from which the data were drawn. This controlled for some differences between sites (e.g., population density, prices, staff qualifications, different methods of serving clients). The original 17 sites were combined into 13 based on location and demographic similarities due to small sample sizes at several sites; only 12 of these sites included individuals seeing non-specialists. Due to perfect prediction, the models dropped up to three sites out of some of the analyses. In the specialist group, two of the sites were dropped in the model looking at the effects of medical skepticism on Diet. In the non-specialist group, one site was dropped for each of the models due to perfect prediction, except for Diet where two sites were dropped. The sites which were dropped were small; in order to keep these sites in the model, the sites could have been combined with other sites. This, however, would have compromised the similarities within sites, thus we allowed the models to drop the sites, meaning that between 2 and 50 people were dropped from different models. This regression was run for each of the ten CAM categories for both groups (specialists and non-specialists).

Differences between responders and non-responders were calculated using two-sample *t*-tests. The responders and non-responders were significantly different on most characteristics. For the non-specialist group, respondents were more often older, unmarried, female, white and had more years of education than participants who did not respond. For the specialist group, respondents had the same characteristics, except there were no statistically significant differences for education and NHAAs.

## RESULTS

Eighty-eight percent of the samples (specialist and non-specialists combined) used some form of CAM for their pain related to musculoskeletal disorders (90% of the specialists and 84% of the non-specialists), with different proportions of the populations using different methods (from 10% of the non-specialist group using special diets to 63% of the specialist group using rubs) (see Table **1**). The mean score on the medical skepticism scale was 2.9, and individuals seeing non-specialists had higher scores than did individuals seeing specialists (3.0 *vs* 2.8, p<0.001), and at least 35% of the sample had average scores on the medical skepticism scale higher than 3. In general, younger individuals (younger than 57 versus 57 and older) used CAM more often, except for vitamins and supplements (not shown). There were significant differences (ranging from between four to nine percentage points) in five of the ten categories of CAM “ever use” (providers, special diets, vitamins, rubs, and mind-body) based on this age dichotomy; medical skepticism was also slightly higher for younger patients (2.9 *vs* 2.8, p<0.001) (not shown).

Both groups consisted of mostly women (77-80%). Almost half of the sample (46-50%) attended some college, graduated from college, or attended a post-graduate program. Participants in both groups were between the ages of 19 and 97. A larger percentage of participants seeing non-specialists was NHAA (19% compared to 14% in the specialist group), with only 3 to 4% of either group in the category of other and multiple races and ethnicities. Seventy-four (specialists) to 77 (non-specialists) percent of the sample worked either in the formal labor force, at home or at school or was retired, and only about a third of either sample was not married. Slightly more than half of both groups reported having good or excellent health. Participants seeing non-specialists had significantly more comorbidities (2.9) than did participants seeing specialists (1.1).

Increased skepticism was associated with the use of one CAM category in the non-specialist group (supplements: OR 1.31 95% CI [1.01 - 1.70]) (see Table **2**). In the specialist group, increased skepticism was associated with the use of six CAM categories, alternative providers (1.26 [1.06 - 1.50]), special diets (1.31 [1.04 - 1.65]), supplements (1.56 [1.32 - 1.85]), rubs (1.21 [1.02 - 1.44]), body treatments (1.49 [1.26 - 1.76]), and mind-body (1.33 [1.12 - 1.58]).

The marginal effects of medical skepticism on the probability of CAM use were positive in all of the models. In both specialist and non-specialist groups, on average, holding all else equal, if a person increased their skepticism from a score of one to a score of five, the probability of any CAM use would increase by almost 90 percentage points. The marginal effects, however, were different for each observation.

Most of the other characteristics were significant in some, but not all, of the models, as is shown by the results from *one* of the models (ever use of CAM) in Table **3** (other models are not shown here). For example, being male was a significant negative predictor of all except for two categories of use of CAM in the specialist group, but was only a significant negative predictor in three of the categories of CAM use for participants seeing non-specialists. For participants seeing non-specialists, having more education was positively related to use of all CAM therapies, but negatively related to the use of rubs, although only a couple of the categories reached levels of significance. The same pattern was seen for participants seeing specialists, although the use of rubs and other body treatments were negatively related to higher levels of education but, again, several categories did not reach levels of significance. Self-reporting as NHAA was a strong predictor of use of special diets (1.87 [1.22 - 2.88]), rubs (1.79 [1.20 - 2.66]), spiritual methods (2.28 [1.58 - 3.28]) and mind-body activity (1.72 [1.19 - 2.48]) for participants seeing specialists. For participants using non-specialists, self-reporting as NHAA was only significantly related to less use of body treatments (0.5 [0.28 - 0.88]). Not working was significantly related to only a few types of CAM use (movement and mind-body activities in non-specialists; supplements in specialists). Being in good or excellent health was not a significant predictor of any use of CAM in the non-specialist group, but was strongly related to the use of seven categories of CAM use in the specialist group, not including vitamins, supplements and movement activity.

## DISCUSSION

Although medical skepticism has been associated with decreased use of conventional methods of care [11], its relationship with CAM use is more tentative. In the CAM outcomes reported here, associations existed between skepticism and use of only one category of CAM in the group seeing non-specialists and six categories in the group seeing specialists.

Thus, medical skepticism does play a role in the use of multiple types of CAM, especially in populations seeing specialists. Our hypothesis is that because patients seeing specialists are usually experiencing more severe manifestations of their illness [15, 16] than patients seeing non-specialists, it could be expected that those seeing specialists might have more skepticism and thus use more forms of CAM.

Medical skepticism was associated with the use of more categories of CAM even though individuals seeing specialists had slightly lower (0.2 difference) medical skepticism scores. Although this may seem counterintuitive, the average medical skepticism scores were both close to three, thus suggesting a high base level of skepticism. In addition, as noted by the marginal effects, increasing medical skepticism greatly increases the probability of CAM use. This finding is in line with our original hypothesis.

The association between medical skepticism and some types of CAM use begs the following questions: 1) What are the consequences of the relationship between medical skepticism and the use of CAM? 2) Are patients substituting CAM for conventional treatment? And if so, is this affecting their health outcomes, and in what direction? 3) Similarly, does medical skepticism affect communication, and inversely, does communication affect medical skepticism? And finally 4) How do these interactions play into the use of CAM?

More knowledge about these areas is important for a variety of reasons. For example, although little is known about provider communication and medical skepticism, research has shown that communication between patients and primary care providers about the use of complementary therapies often depends on the actions of the provider [9, 20, 21]. Although fear of disapproval was rarely cited as a reason for not discussing CAM use in one study, the authors found that one of the most common reasons for not disclosing CAM use was that the physician had not requested that information [9]. This evidence suggests that providers may not have full information about individual patterns of health seeking behaviors.

The ramifications of these communication gaps are largely unknown, but could be important predictors of health outcomes. For example, interactions between CAM use and conventional therapy are possible; thus the provider needs to know about the patients’ use of alternative therapies. But the goal should not only be to avoid interactions between medications and CAM therapies; providers should also want to enhance the care of their patients. Therefore, conversing about the latest research on conventional therapies and on CAM could be helpful in decision-making. In addition, patients with high levels of skepticism may need even more information and education about therapies—and the topics of conversation may need to vary from the status quo. Although more research is needed in this area, it could be hypothesized that different types of education and communication about therapies may be more appropriate for skeptical individuals. For example, skeptical individuals may desire to know more about the side-effects and limitations of conventional medication. Frank conversations about these topics may enhance levels of trust for skeptical patients.

Another reason for providers to pay attention to levels of medical skepticism is that consumers with more skepticism concerning medical care [22] rated the medical care they were given more negatively than non-skeptical consumers [23]. Consumer evaluations are often used to assess performance of physicians [24], thus Crofton, Lubalin, and Darby [22] noted that policymakers and providers need to consider the degree to which they should attempt to satisfy skeptical consumers while also targeting skeptical consumers with educational efforts to explain the benefits of medical care. The findings from our study show that skepticism is an issue for a significant proportion of this population. Therefore, more knowledge about relationships between skepticism, CAM use and, eventually, health outcomes, could be a stepping stone to greater patient satisfaction, especially if providers and policymakers were to make a concerted effort to address the issue of skepticism *via* communication and education.

**Limitations**. Several limitations should be noted. First, income and area of residence (rural versus urban) were not included in the model. Employment was used as a proxy for income. Future research should look into including these in similar models.

The outcome relates to the survey question as to whether someone used CAM “specifically for your arthritis or joint symptoms”. If a participant misread the question, then she might have answered erroneously, indicating whether she has ever used CAM for any reason. Although 88% of the population noted they used CAM for their musculoskeletal disorders, which is higher than the 40-60% noted in other studies [8–10], our study included prayer. The numbers reported in another study which also included prayer as a form of CAM were similar to those found here [25], and Quandt, Chen, Grzywacz, Bell, Lang, and Arcury [10] noted that their findings might be lower because they did not include prayer. Future research should validate the questionnaire to ensure that participants fully understand the meaning of these types of questions.

A similar limitation arises from the use of the question about whether someone used CAM “specifically for your arthritis or joint symptoms”. RA, OA, FM and chronic joint symptoms are all very different diseases with varying manifestations and comorbidities. These types of differences between diseases affect health care-seeking behaviors and could also affect medical skepticism and CAM use. Future work is necessary to better define these relationships for different forms of musculoskeletal disorders.

In addition, the effect of disease duration on medical skepticism could be important in determining CAM use. We did not have adequate numbers to look at this question but future research should parse out the differences between duration, medical skepticism and use of CAM.

Finally, this study was based on a cross-sectional survey. This limited our ability to rule out that the observed associations are not due to unobserved time-invariant individual characteristics. Future research should look into possible associations between CAM and medical skepticism using longitudinal data.

Although the limitations noted here are important for future research in this area, we do not believe that they bias the model significantly. In particular, the models are based on large sample sizes and the findings agree with other studies in the area.

## CONCLUSIONS

With many individuals reporting high levels of medical skepticism and high rates of CAM use, failing to address these areas would be a lost opportunity to better engage patients in their own care. Goals should be to improve communication based on the skepticism of the individual, avoid interactions between CAM therapies and conventional medications, and enhance a patient’s care regimen by educating them about the latest research and ways in which conventional medicine can be combined with complementary and alternative therapies.

## ETHICAL APPROVAL

This study was approved by the UNC School of Medicine Institutional Review Board.

## Figures and Tables

**Table 1:** Characteristics of a Sample of Patients with Muscu-loskeletal Disorders from North Carolina in 2001 Seeing Specialists and Non-Specialists

	Specialists % or Mean (sd) n=1,344	Non-Specialists % or Mean (sd) n=724
**Types of CAM Use**		
Ever used CAM	90	84
Therapist	36	35
Diet	16	10
Vitamin	30	18
Supplement	38	26
Rub	63	60
Body Treatment	36	24
Movement	30	23
Spirit	50	38
Mind-Body	37	25
**Medical Skepticism**	2.79 (0.71)	3.01 (0.72)
Demographics		
Sex (% Male)	20	23
Education (% College or more)	50	46
Education (% Missing)	3	2
Age	58 (13)	54 (15)
Race and Ethnicity		
(% Non-Hispanic African	14	19
American (NHAA))		
Race and Ethnicity (% Other and multiple races)	3	4
Race and Ethnicity (% Missing)	5	1
Employment (% Unemployed)	26	23
Employment (% Missing)	5	3
Marital Status (% Unmarried)	33	39
Marital Status (% Missing)	2	1
General Health Status (% Good and excellent health)	51	57
Number of Comorbidities	1.1 (1.9)	2.9 (1.9)

SD=Standard Deviation.

**Table 2:** Adjusted† Odds Ratios on the Effects of Medical Skepticism on Ever Use of a Variety of CAM Types in Patients with Musculoskeletal Disorders in the Specialist and Non-Specialist Groups

	Specialist OR (95% CI)	Non-Specialist OR (95% CI)
Ever used CAM	1.22 (0.91 - 1.62)	1.30 (0.96 - 1.76)
Therapist	1.26 (1.06 - 1.50)**	1.17 (0.91 - 1.49)
Diet	1.31 (1.04 - 1.65)*	1.45 (0.96 - 2.21)
Vitamin	1.09 (0.91 - 1.29)	1.23 (0.91 - 1.65)
Supplement	1.56 (1.32 - 1.85)**	1.31 (1.01 - 1.70)*
Rub	1.21 (1.02 - 1.44)*	1.21 (0.96 - 1.51)
Body Treatment	1.49 (1.26 - 1.76)**	1.21 (0.93 - 1.57)
Movement	0.99 (0.83 - 1.17)	1.13 (0.88 - 1.46)
Spirit	0.96 (0.81 - 1.13)	1.06 (0.84 - 1.34)
Mind-Body	1.33 (1.12 - 1.58)**	1.13 (0.87 - 1.46)

† From a site-level (13 sites for specialists and 12 sites for non-specialists) fixed-effects logistic regression model adjusted for sex, education, age, race and ethnicity, employment, marital status, and health status. In the specialist group, 2 of the sites were dropped due to perfect prediction in Diet. In the non-specialist group, 1 site was dropped for each of the models due to perfect prediction, except for Diet where 2 sites were dropped. All specialist samples included 1,313 individuals, except for Diet, which had 1,278 individuals. All non-specialist samples included 710 individuals, except for Diet, which had 662 individuals, and Body Treatments, which had 702 individuals. The models controlled for differences at the site level using fixed effects for the sites.

* Statistically significant at the 5 percent level.

** Statistically significant at the 1 percent level. OR = Odds Ratio. CI = Confidence Interval.

**Table 3.** Adjusted† Odds Ratios on the Effects of Medical Skepticism on Ever Use of any Type of CAM in Patients with Musculoskeletal Disorders

	Specialist OR (95% CI)	Non-Specialist OR (95% CI)
Medical Skepticism	1.22 (0.91 - 1.62)	1.30 (0.96 - 1.76)
Sex (Male)	0.61 (0.39 - 0.95)*	0.77 (0.48 - 1.24)
Education (College or more)	1.29 (0.87 - 1.90)	1.13 (0.72 - 1.77)
Age	0.99 (0.97 - 1.00)	1.00 (0.98 - 1.01)
Race and Ethnicity (Non- Hispanic African American (NHAA))	1.22 (0.68 - 2.18)	1.27 (0.69 - 2.33)
Race and Ethnicity (Other and multiple races)	0.72 (0.22 - 2.32)	2.41 (0.54 - 10.80)
Employment (Unem- ployed)	1.05 (0.66 - 1.67)	1.29 (0.75 - 2.22)
Marital Status (Unmarried)	0.71 (0.46 - 1.09)	0.86 (0.55 - 1.36)
General Health Status		
(Good and excellent health)	0.60 (0.40 - 0.90)*	1.12 (0.68 - 1.86)
Number of Comorbidities	0.96 (0.83 - 1.11)	1.12 (0.98 - 1.28)

† For site-level (13 sites in specialist and 11 in non-specialist) fixed-effects logistic regression model adjusting for the other variables listed in the tables. There were 1,313 observations in the specialist group and 710 in the non-specialist group. The dummy variable adjustment was used for missings on education, race and ethnicity, employment and marital status. The odds ratios on those variables are not shown here.

* Statistically significant at the 5 percent level.

** Statistically significant at the 1 percent level. OR = Odds Ratio. CI = Confidence Interval.

## ABBREVIATIONS

CAM: Complementary and Alternative Medicine
CJS: Chronic joint symptoms
FM: Fibromyalgia
NHAA: Non-Hispanic African American
NHC: Non-Hispanic Caucasian
OA: Osteoarthritis
RA: Rheumatoid arthritis
